# Enhanced Oilfield-Produced-Water Treatment Using Fe^3+^-Augmented Composite Bioreactor: Performance and Microbial Community Dynamics

**DOI:** 10.3390/bioengineering12070784

**Published:** 2025-07-19

**Authors:** Qiushi Zhao, Chunmao Chen, Zhongxi Chen, Hongman Shan, Jiahao Liang

**Affiliations:** 1 College of Chemical Engineering and Environment, China University of Petroleum-Beijing, Beijing 102249, China; zhaoqiushi@petrochina.com.cn (Q.Z.); chenzhongxi@petrochina.com.cn (Z.C.); 2 Daqing Oilfield Design Institute Co., Ltd., Daqing 163712, China; shanhongman@petrochina.com.cn; 3 Key Laboratory of Petrochemical Pollution Control of Guangdong Higher Education Institutes, School of Environmental Science and Engineering, Guangdong University of Petrochemical Technology, Maoming 525000, China

**Keywords:** biodegradation, chemical-flooding-produced water, oilfield wastewater, microbial community

## Abstract

The presence of recalcitrant organic compounds in oilfield-produced-water poses significant challenges for conventional biological treatment technologies. In this study, an Fe^3+^-augmented composite bioreactor was developed to enhance the multi-pollutant removal performance and to elucidate the associated microbial community dynamics. The Fe^3+^-augmented system achieved efficient removal of oil (99.18 ± 0.91%), suspended solids (65.81 ± 17.55%), chemical oxygen demand (48.63 ± 15.15%), and polymers (57.72 ± 14.87%). The anaerobic compartment served as the core biotreatment unit, playing a pivotal role in microbial pollutant degradation. High-throughput sequencing indicated that Fe^3+^ supplementation strengthened syntrophic interactions between iron-reducing bacteria (*Trichococcus* and *Bacillus*) and methanogenic archaea (*Methanobacterium* and *Methanomethylovorans*), thereby facilitating the biodegradation of long-chain hydrocarbons (e.g., eicosane and nonadecane). Further metabolic function analysis identified long-chain-fatty-acid CoA ligase (EC 6.2.1.3) as a key enzyme mediating the interplay between hydrocarbon degradation and nitrogen cycling. This study elucidated the ecological mechanisms governing Fe^3+^-mediated multi-pollutant removal in a composite bioreactor and highlighted the potential of this approach for efficient, sustainable, and adaptable management of produced water in the petroleum industry.

## 1. Introduction

As most global oilfields increasingly enter mid-to-late production stages, a significant portion of crude oil remains trapped within reservoir pores due to capillary, viscous, gravitational, and other hydrodynamic effects [1]. Chemical Enhanced Oil Recovery (CEOR) technologies—which inject chemical flooding agents to increase solution viscosity, expand sweep volume, and regulate fluid flow—have been established as the principal, economically viable strategies for recovering residual oil, with widespread application in countries such as China, the United States, and Canada [2,3]. However, the chemical flooding process generates chemical-flooding-produced water (CFPW) characterized by high concentrations of polymeric agents, emulsified oil droplets, and a diversity of complex organics [4]. These attributes result in markedly increased water viscosity and enhanced emulsion stability, rendering the composition of produced water increasingly complex and significantly challenging its effective treatment [5,6].

Traditional physicochemical processes, including coagulation–flocculation, air flotation, advanced oxidation, and membrane separation, have long been used for treating CFPW due to their rapid pollutant removal and reliability. However, these approaches often incur high operational costs, generate secondary pollutants, and show limited efficacy in degrading persistent organic compounds [7,8,9,10,11]. In contrast, biological treatment offers a promising green alternative for produced water management, known for its environmental compatibility, cost-effectiveness, and sustainability [12]. Biotechnologies such as fixed-bed hybrid bioreactors (FBHBRs) [13], biological-contact-oxidation reactors (BCORs) [14], membrane-aerated biofilm reactors (MABRs), and immobilized biological systems [15] have shown promising potential for oilfield-produced water treatment. By facilitating the formation of biofilms or immobilizing functional microorganisms, these technologies enhance the metabolic diversity and robustness of microbial communities, thereby enabling the effective degradation of complex organic pollutants present in the produced water. This capability is particularly crucial for maintaining high treatment efficiency under fluctuating operational conditions. Nevertheless, the inherently low biodegradability of CFPW remains a significant bottleneck that limits the overall efficiency of biological processes [16]. Thus, enhancing the biodegradation capacity is critical for advancing oilfield-produced-water treatment technologies.

Iron-based materials have recently attracted significant attention due to their multifunctional roles in environmental remediation [17]. Besides serving as an essential trace element for microbial growth, iron species facilitate pollutant degradation through redox pathways. For instance, zero-valent iron (Fe^0^) undergoes corrosion to continuously release H_2_ and Fe^2+^, supplying both electrons and hydrogen to methanogenic archaea, thereby promoting direct interspecies electron transfer (DIET) between syntrophic acetogens and methanogens, and methanogenesis [18]. Iron oxides, such as Fe_3_O_4_, can act as electron shuttles to further accelerate DIET and enhance the co-metabolic degradation of recalcitrant organics [19]. Furthermore, Fe^2+^/Fe^3+^ cycling activates persulfate and hydrogen peroxide to generate highly reactive SO_4_·^−^ and ·OH radicals, sustaining the advanced oxidation of organics [20,21]. Numerous studies have confirmed that iron-based materials can substantially improve microbial degradation efficiency towards organic contaminants [22,23,24,25]. Notably, microbial processes enhanced by Fe^3+^ reduction have recently shown great promise, with Fe^3+^ addition substantially improving chemical oxygen demand (COD) and total nitrogen (TN) removal in conventional A^2^O systems [26,27,28,29]. These findings suggest that the integration of iron reduction in CFPW biotreatment processes may significantly augment pollutant-removal efficiencies.

This study aimed to (1) systematically evaluate the performance and application potential of an Fe^3+^-augmented composite bioreactor treating CFPW; (2) elucidate the interrelationship between pollutant degradation and iron ion transformation under Fe cycling; and (3) characterize the impacts of Fe cycling on microbial community structure and function. The results provided critical scientific support for the development and application of Fe-cycling-based biotreatment technologies for oilfield-produced water, while offering innovative insights for tackling the challenges associated with recalcitrant organic biodegradation in oilfield wastewater.

## 2. Materials and Methods

### 2.1. Oilfield-Produced Water

The actual oilfield-produced water used in this study was collected from the influent of a wastewater treatment facility at Daqing Oilfield. Detailed physicochemical properties are listed in Table 1.

### 2.2. Experimental Setup and Operating Procedure

**Figure 1:**
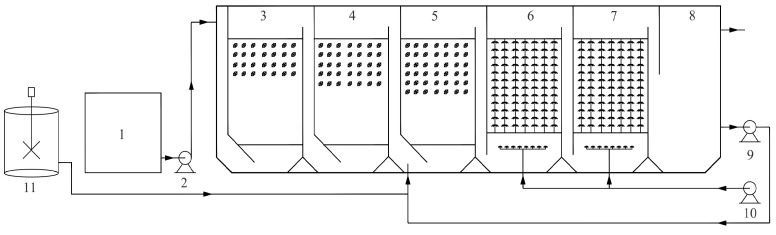
Schematic diagram of the experimental setup: (1) influent tank; (2) lift pump; (3) Anaerobic Zone 1; (4) Anaerobic Zone 2; (5) Anoxic Zone; (6) Aerobic Zone 1; (7) Aerobic Zone 2; (8) sedimentation zone; (9) recirculation pump; (10) aeration pump; (11) chemical dosing tank.

The composite bioreactor used in this study consisted of two anaerobic zones, an anoxic zone, two aerobic zones, and a settling zone (Figure 1). Suspended biofilm carriers were installed in both the anaerobic and anoxic zones to promote microbial attachment and growth, with filling ratios of 30% and 35% for Anaerobic Zones 1 and 2, respectively, and 40% for the anoxic zone. In the aerobic zones, a combination of packing materials was positioned above the aeration system to enhance biofilm formation. The effective working volume of each reaction zone (Anaerobic Zone 1, Anaerobic Zone 2, Anoxic Zone, Aerobic Zone 1, and Aerobic Zone 2) was 48 L, resulting in a total effective reactor volume of 240 L (excluding the settling chamber).

During operation, the overall hydraulic retention time (HRT) of the composite bioreactor was maintained at 16 h. The oilfield-produced water flowed sequentially through the anaerobic, anoxic, and aerobic zones. Effluent from the aerobic zone was recycled to the anoxic zone at a ratio of 100%. Dissolved oxygen (DO) in the aerobic zone was maintained at 2–3 mg/L. To optimize nutrient balance and assess the effect of Fe^3+^ augmentation, the influent was supplemented with ferric chloride (Fe^3+^ concentration: 100 mg/L), urea (total nitrogen: 5 mg/L), and monopotassium phosphate (total phosphorus: 2 mg/L).

### 2.3. Analytical Methods

#### 2.3.1. Physicochemical Analysis

COD was determined using the potassium dichromate titration method [30]. Oil content and suspended solids (SS) were measured according to the China Petroleum Industry Standard SY/T 5329-2022. TN was quantified via alkaline potassium persulfate digestion followed by UV spectrophotometry (HJ 636-2012), and ammonia nitrogen (NH_4_^+^-N) was determined using Nessler’s reagent spectrophotometry (HJ 535-2009). UV absorbance at 254 nm (UV_254_) was measured as a supplementary indicator for assessing the presence of organic compounds with conjugated double bonds and aromatic structures, in accordance with the procedure specified in DB 37/T 4149-2020. Total iron and ferrous ion (Fe^2+^) concentrations were analyzed using phenanthroline spectrophotometry (HJ/T 345-2007).

#### 2.3.2. Organic Composition Analysis

After 45 days of operation, water samples were collected to evaluate the removal of refractory organic compounds. Analytical measurements were conducted using a gas chromatograph–mass spectrometer (GC-MS, 7890A-5975C, Agilent Technologies Inc., Santa Clara, CA, USA), which was fitted with a DB-5MS capillary column (30 m × 0.25 mm × 0.25 μm; Agilent Technologies Inc., Santa Clara, CA, USA). Target analytes were isolated from the aqueous matrices via a liquid–liquid extraction protocol employing HPLC-grade dichloromethane as the solvent. The column oven was initially held at 80 °C for 1 min, subsequently ramped to 150 °C at a gradient of 7 °C min^−1^. The thermal program then increased the oven temperature to 300 °C at a rate of 10 °C min^−1^, with a final isothermal hold at 300 °C for 5 min. High-purity helium served as the carrier gas at a constant flow rate of 1.0 mL min^−1^, and 1.0 μL of each extract was injected for analysis. The mass spectrometer operated in the electron ionization (EI) mode, with a signal-to-noise ratio of 65:1 and a data acquisition rate of 2 Hz. Spectral resolution was maintained at 0.6 amu (FWHM) using a quadrupole mass analyzer (Agilent Technologies Inc., Santa Clara, CA, USA). Compound identification was achieved by comparing the acquired spectra to entries in the NIST08 mass spectral database. 

#### 2.3.3. Microbial Community Analysis

High-throughput sequencing was employed to characterize the bacterial and methanogenic archaeal communities in each reactor unit. At the end of the experimental period, biofilm samples were collected from Anaerobic Zone 1 (AN1), Anaerobic Zone 2 (AN2), Anoxic Zone (ANO), Aerobic Zone 1 (O1), and Aerobic Zone 2 (O2). Genomic DNA was extracted using the E.Z.N.A.^®^ Soil DNA Kit (Omega Bio-tek, Inc., Norcross, GA, USA). The V3-V4 hypervariable regions of the 16S rRNA gene were amplified using primers 338F (5′-ACTCCTACGGGAGGCAGCAG-3′) and 806R (5′-GGACTACHVGGGTWTCTAAT-3′). Sequencing was performed on the Illumina MiSeq platform (Majorbio Bio-Pharm Technology Co., Ltd., Shanghai, China), and bacterial community composition was analyzed via the Majorbio Cloud Platform (https://cloud.majorbio.com (accessed on 11 October 2023)).

#### 2.3.4. Metagenomic Analysis

Metagenomic sequencing was performed using the Illumina HiSeq 4000 platform (Illumina Inc., San Diego, CA, USA), generating 223,675,084 raw reads from 5 samples. A non-redundant gene catalog comprising 1,055,539 genes with an average sequence length of 623.63 bp was constructed. Gene catalog sequences were aligned to the KEGG GENES database using BLASTP (version 2.2.28+, http://blast.ncbi.nlm.nih.gov/Blast.cgi (accessed on 17 October 2023)) with an e-value threshold of 1 × 10^−5^. Functional annotation was performed using KOBAS 2.0 (KEGG Orthology-Based Annotation System, Beijing, China). All bioinformatics analyses, including data processing and annotation, were conducted on the Majorbio Cloud Platform (https://cloud.majorbio.com (accessed on 17 October 2023)).

## 3. Results and Discussion

### 3.1. Pollutant Removal Performance of Fe^3+^-Augmented Bioreactor

**Figure 2:**
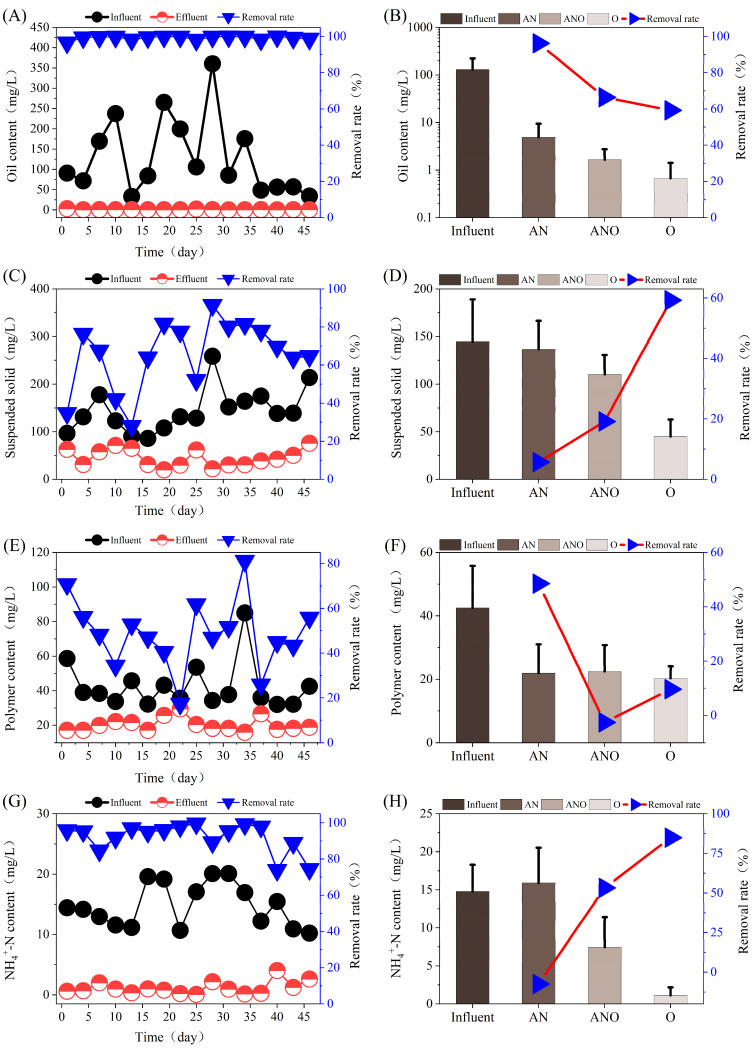
Removal performance of oil content, SS, and polymers in the Fe^3+^-augmented composite bioreactor: (**A**) overall oil content removal; (**B**) oil content removal across treatment units; (**C**) overall SS removal; (**D**) SS removal across treatment units; (**E**) overall polymer removal; (**F**) polymer removal across treatment units; (**G**) overall NH_4_^+^-N removal; (**H**) NH_4_^+^-N removal across treatment units.

Produced water from chemically enhanced oil recovery operations poses considerable treatment challenges due to its high content of emulsified hydrocarbons, SS, and refractory polymers. The treated oilfield-produced water is primarily intended for reinjection, with oil content and SS serving as critical quality parameters for reinjection suitability [31]. As shown in Figure 2A,B, the composite bioreactor demonstrated excellent removal of petroleum hydrocarbons, achieving an average oil-removal efficiency of 99.18 ± 0.91%, while effluent oil concentrations consistently remained below 1 mg/L, meeting reinjection standards. Notably, the anaerobic unit served as the primary functional zone for oil degradation, with an average removal efficiency of 96.22%. Regarding SS removal (Figure 2C,D), the overall removal efficiency reached 65.81 ± 17.55%.

In addition to conventional pollutants, residual polymers—particularly hydrolyzed polyacrylamide (HPAM), commonly applied in enhanced oil recovery—posed significant treatment challenges due to their high molecular weight, structural complexity, and emulsifying properties [32]. As shown in Figure 2E,F, the removal efficiency of polymers reached an average of 48.63 ± 15.15%, with the anaerobic unit identified as the major zone for polymer removal (mean removal: 48.49%). To assess the biodegradation of organic nitrogen in polymers, NH_4_^+^-N was selected as an indicator. As shown in Figure 2G,H, a total NH_4_^+^-N removal efficiency of 92.40% was achieved. The NH_4_^+^-N concentrations in the anaerobic effluent exceeded those in the influent, suggesting that microbial activity in the anaerobic unit successfully converted organically bound nitrogen from polymers into ammonia. This observation provided indirect evidence for the microbial-mediated depolymerization of nitrogenous macromolecules, potentially including HPAM, and highlights the functional role of Fe^3+^ in accelerating redox-driven bioconversions. The superior performance of the system was largely attributed to the bioaugmentation effect conferred by the Fe^3+^ addition, which served as an effective electron acceptor and stimulated the proliferation and activity of iron-reducing bacteria (FeRB) [33]. By enhancing anaerobic hydrolysis and acidogenesis, Fe^3+^ introduction facilitated the degradation of petroleum hydrocarbons and high-molecular-weight polymers. The composite bioreactor demonstrated superior oil removal efficiency (96.2%) compared to previously reported systems lacking Fe^3+^ augmentation, which exhibited removal efficiencies in the range of 73.97% to 86.3% [34,35]. This significant improvement confirms the synergistic benefits of Fe^3+^-mediated bioaugmentation in enhancing oil-degradation performance. These findings highlight the addition of Fe^3+^ as a viable strategy for enhancing bioreactor performance in treating refractory industrial wastewaters [28]. The Fe^3+^-augmented composite bioreactor offered a promising technological solution for the efficient treatment of oilfield-produced water.

**Figure 3:**
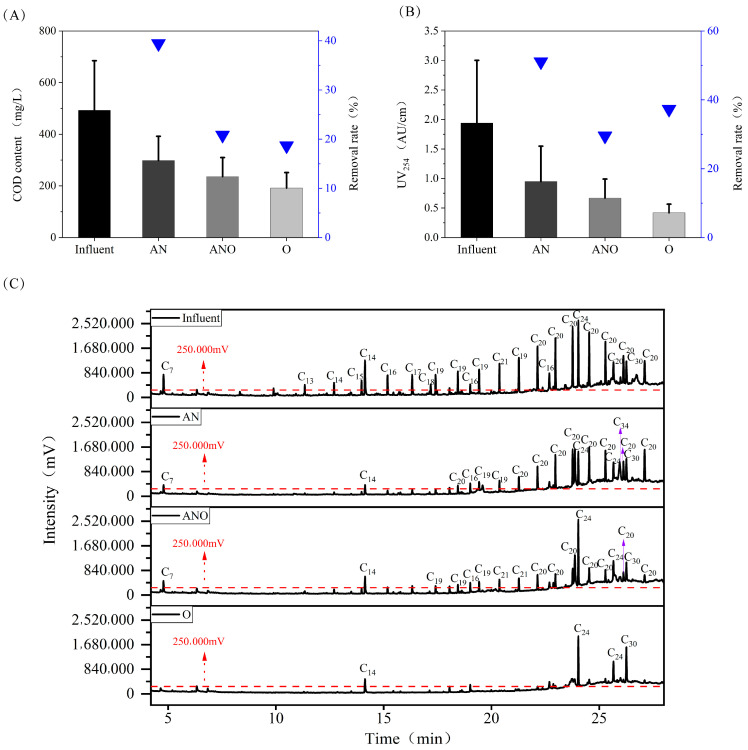
Organic matter removal performance: (**A**) COD removal; (**B**) UV_254_ reduction; (**C**) GC-MS spectrum of organic pollutants.

As illustrated in Figure 3A, the composite bioreactor achieved an average COD removal efficiency of 57.72 ± 14.87%, with the anaerobic compartment accounting for 39.45% of the total removal, thereby functioning as the primary zone for organic mineralization. UV absorbance at 254 nm (UV_254_) is widely recognized as a sensitive indicator for humic-like substances and aromatic organic compounds in aquatic environments [36]. In the anaerobic zone, the UV absorbance decreased markedly from 1.94 ± 1.06 AU/cm in the influent to 0.95 ± 0.60 AU/cm, corresponding to a 51.09% reduction. The anoxic and aerobic units exhibited absorbance reductions of 29.51% and 37.30%, respectively. These findings demonstrate that the Fe^3+^-enhanced biological system, particularly the anaerobic unit, facilitated the efficient microbial degradation of humic-like substances and aromatic organic compounds.

GC-MS analysis (Figure 3C) revealed the presence of 75 organic contaminants in the influent, predominantly long-chain alkanes and alkenes (C7–C30), such as eicosane (32.62%), nonadecane (9.71%), phthalic acid, di(2-propylpentyl) ester (6.85%), 2,4-di-tert-butylphenol (4.39%), squalene (3.16%), heneicosane (2.69%), and heptadecane (2.32%). To systematically assess the degradation performance across the different reactor compartments, organic pollutants exhibiting peak intensities above 250,000 mV were selected as target analytes. Figure 3C shows a clear and progressive reduction in the number of analyte peaks above the 250,000 mV threshold along the treatment continuum from anaerobic to anoxic and, subsequently, to aerobic stages. This sequential decline underscored an effective stepwise attenuation of recalcitrant organic pollutants. Following the anaerobic phase, the total count of such peaks diminished to 19, signaling substantial contaminant removal at this stage of the treatment process. Notably, predominant compounds such as eicosane, phthalic acid di(2-propylpentyl) ester, nonadecane, squalene, and 2,4-di-tert-butylphenol showed substantial reductions in peak intensity, while easily biodegradable compounds, including 2-methyl-5-propyl-nonane and tetradecane, diminished below the selected threshold, indicating that the anaerobic process facilitated the breakdown of recalcitrant hydrocarbons into lower-molecular-weight intermediates [37]. Within the anoxic zone, structural destabilization of high-molecular-weight organics promoted their aggregation and recombination, resulting in the emergence or increased intensities of certain compounds. For example, phthalic acid di(2-propylpentyl) ester exhibited a higher peak intensity than its initial level, becoming a primary residual contaminant alongside eicosane and terephthalic acid 2-ethylhexyl octyl ester. Upon aerobic treatment, both the number and overall intensity of high-abundance organic peaks decreased considerably, with only four compounds—phthalic acid di(2-propylpentyl) ester, squalene, 1,3-benzenedicarboxylic acid bis(2-ethylhexyl) ester, and 2,4-di-tert-butylphenol—remaining above 250,000 mV. Squalene exhibited a higher peak intensity in the aerobic effluent than in the influent. The reaction of small-molecule degradation products with destabilized macromolecules may be responsible for the formation of recalcitrant compounds (>C20). Overall, the Fe^3+^-augmented composite bioreactor demonstrated pronounced efficiency in eliminating residual petroleum-derived organics from oilfield-produced water, with particularly high removal efficiencies observed for alkanes such as eicosane and nonadecane. Notably, the marked reduction in persistent compounds, exemplified by phthalic acid di(2-propylpentyl) ester, underscores the crucial role of Fe^3+^ in stimulating microbial bio-oxidation pathways targeting structurally complex organic pollutants. These findings not only validate the enhanced degradative capability of the Fe^3+^-mediated biological system for conventional hydrocarbon contaminants but also highlight their potential for the effective removal of recalcitrant organic species in oilfield-produced water.

### 3.2. Microbial Community Structure

#### 3.2.1. Bacterial Community Analysis

**Figure 4:**
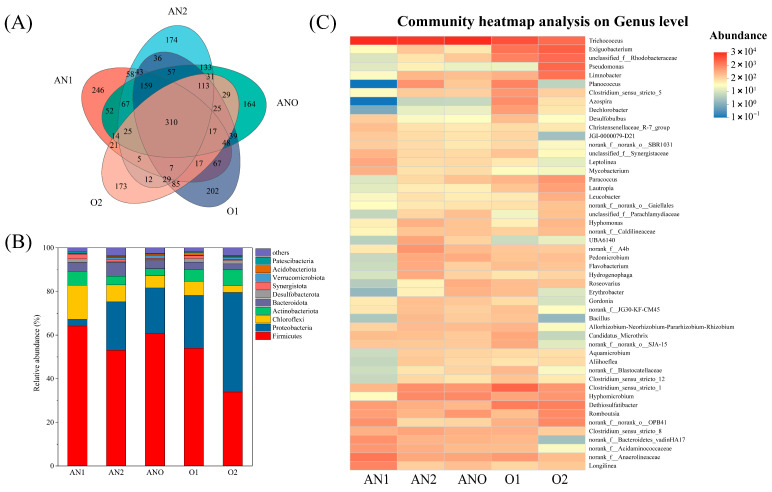
Bacterial community composition: (**A**) Venn diagram; (**B**) phylum level; (**C**) genus level.

The bacterial diversity indices of all samples are summarized in Table 2. Sequencing coverage for each of the six samples exceeded 99.9%, affirming the comprehensiveness and representative quality of the community profiling. As shown in Figure 4A, a total of 310 operational taxonomic units (OTUs) were commonly observed across all bioreactor zones, with Anaerobic Zone 1 (AN1) exhibiting the highest number of unique OTUs. The elevated bacterial diversity not only enhanced the system’s capacity for the degradation of a broad range of pollutants but also improved its resilience and adaptability to toxic and high-strength contaminant shocks. A progressive decline in the relative abundance of Firmicutes and Chloroflexi was observed from anaerobic through anoxic to aerobic stages, accompanied by a marked enrichment of Proteobacteria (Figure 4B). The relative abundances of Proteobacteria across bioreactor compartments were 3.13% (AN1), 22.25% (AN2), 20.76% (ANO), 24.23% (O1), and 45.62% (O2), respectively. This distribution pattern suggested that Proteobacteria not only persisted through all stages but that their abundance was positively correlated with increased dissolved oxygen concentrations, culminating in their dominance in Aerobic Zone 2. The eventual predominance of Proteobacteria could be ascribed to their exceptional metabolic flexibility and ecological adaptability, which enable them to exploit a broad spectrum of organic substrates and efficiently perform aerobic metabolic processes. Additionally, many members of the Proteobacteria phylum are known to play key roles in nutrient cycling, including ammonia oxidation and denitrification, further supporting their competitive advantage under aerobic conditions [38].

As shown in Figure 4C, *Trichococcus* was the dominant genus in both the anaerobic and anoxic zones. This genus is well-adapted to anaerobic and anoxic environments and is recognized as a key fermentative bacterium with the capability to metabolize complex organic substrates into acetate and formate [39,40]. Moreover, *Trichococcus* has been identified as a pivotal functional genus involved in the biodegradation of high-molecular-weight polymers [41]. In the aerobic compartments, facultative anaerobes such as *Clostridium sensu stricto* 1 and *Exiguobacterium* became prominent, mediating the hydrolytic acidification of organic matter and petroleum hydrocarbons [41,42,43]. The presence of these bacteria under aerobic conditions was attributable to the stratified oxygen gradients within the biofilm, which created hypoxic microenvironments conducive to anaerobic metabolism. *Pseudomonas* was also abundant in the aerobic zone. The genus *Pseudomonas* demonstrated multifunctionality during wastewater treatment. Firstly, it was capable of synthesizing biosurfactants and accumulating intracellular poly-β-hydroxybutyrate (PHB) granules, thereby promoting the degradation of petroleum hydrocarbons, denitrification, and heterotrophic nitrification [44,45]. Secondly, it actively participated in Fe-mediated nitrogen cycling, particularly through the oxidation of NH_4_^+^-N [46,47].

Additionally, Fe^3+^ introduction enriched FeRB, such as *Trichococcus* [48], *Bacillus* [49], *Clostridium sensu stricto 1* [50], and *Desulfobulbus* [51]. *Trichococcus* emerged as the dominant genus in the AN1, AN2, and ANO compartments (Figure 4C). This genus facilitated the reduction of Fe^3+^ to Fe^2+^ while simultaneously enhancing the degradation of organic pollutants through microbial electron transfer processes. *Clostridium sensu stricto 1* gradually increased from AN2 to ANO and remained prevalent in O1, likely due to localized anaerobic/oxic microniches within the aerobic biofilm carriers, consistent with the detection of Fe^2+^ in aerobic effluent (Section 3.4). *Bacillus* and *Desulfobulbus*, though present at lower abundances, may contribute to ancillary processes such as sulfate reduction.

In addition, several iron-oxidizing bacteria (FeOB) were detected, including *unclassified f__Rhodobacteraceae* [52], *Azospira* [53], and *Aliihoeflea* [54]. Among these, *unclassified __Rhodobacteraceae* dominated the aerobic zones, playing a central role in mediating Fe^2+^ oxidation to Fe^3+^. Notably, *Pseudomonas* and *Paracoccus*, which possessed dual capabilities for iron reduction [55,56] and iron oxidation [57], were detected at low abundance in the anaerobic zones, where they likely played auxiliary roles in Fe^3+^ reduction. In contrast, their abundance increased markedly in the aerobic zones, where they were the predominant taxa responsible for Fe^2+^ oxidation. These findings reveal a distinct ecological succession and division of labor among key iron-cycling microorganisms in Fe^3+^-augmented systems. Overall, the Fe^3+^-augmented bioreactor fostered a targeted and dynamic microbial community structure that strongly supported the efficient removal of recalcitrant contaminants and the synergistic coupling of multiple biogeochemical processes.

#### 3.2.2. Archaeal Community Analysis

**Figure 5:**
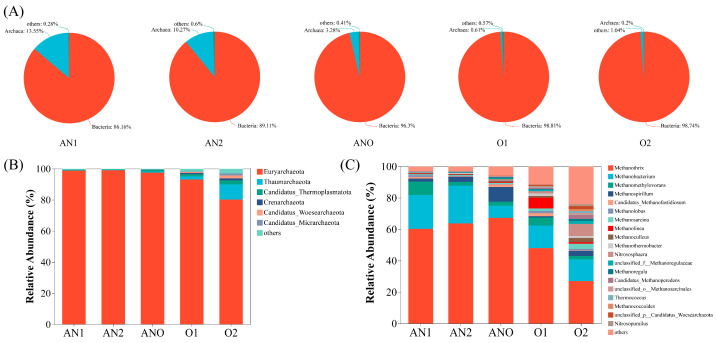
Archaeal community composition: (**A**) archaeal distribution; (**B**) phylum level; (**C**) genus level.

In anaerobic environments, archaea and bacteria synergistically interact to facilitate petroleum hydrocarbon biodegradation [58,59]. As shown in Figure 5A,B, archaeal communities were predominantly distributed in the anaerobic and anoxic units. Euryarchaeota were the principal functional phylum throughout the bioreactor zones. Methanogens, including *Methanothrix*, *Methanobacterium*, *Methanomethylovorans*, and *Methanospirillum*, were ubiquitously distributed across all treatment units, demonstrating their metabolic versatility and syntrophic potential. Integrated analysis of pollutant removal efficiency (Figure 2) and the spatial distribution of microbial communities across bioreactor compartments revealed significant co-occurrence between methanogenic archaea and bacteria, particularly in the anaerobic and anoxic zones. This spatial association suggests functional complementarities that are critical for pollutant removal processes [60]. Specifically, *Methanothrix* and *Methanomethylovorans* in the anaerobic/anoxic units were known to facilitate DIET, therefore improving the efficiency of anaerobic digestion [61,62]. *Methanobacterium*, widely reported in petroleum-contaminated environments, demonstrated hydrocarbon-degrading potential [63,64]. Integrating the results of the oil removal (Figure 2A) and organic degradation characteristics (Figure 3C), it was inferred that *Methanobacterium* and *Methanomethylovorans* likely established syntrophic partnerships with dominant bacterial genera such as *Trichococcus* in the anaerobic zones, thereby enhancing the removal of petroleum hydrocarbons. This cooperative interaction among archaeal and bacterial communities supported the enhanced treatment performance observed in the Fe^3+^-augmented bioreactor.

### 3.3. Metabolic Function Analysis

**Figure 6:**
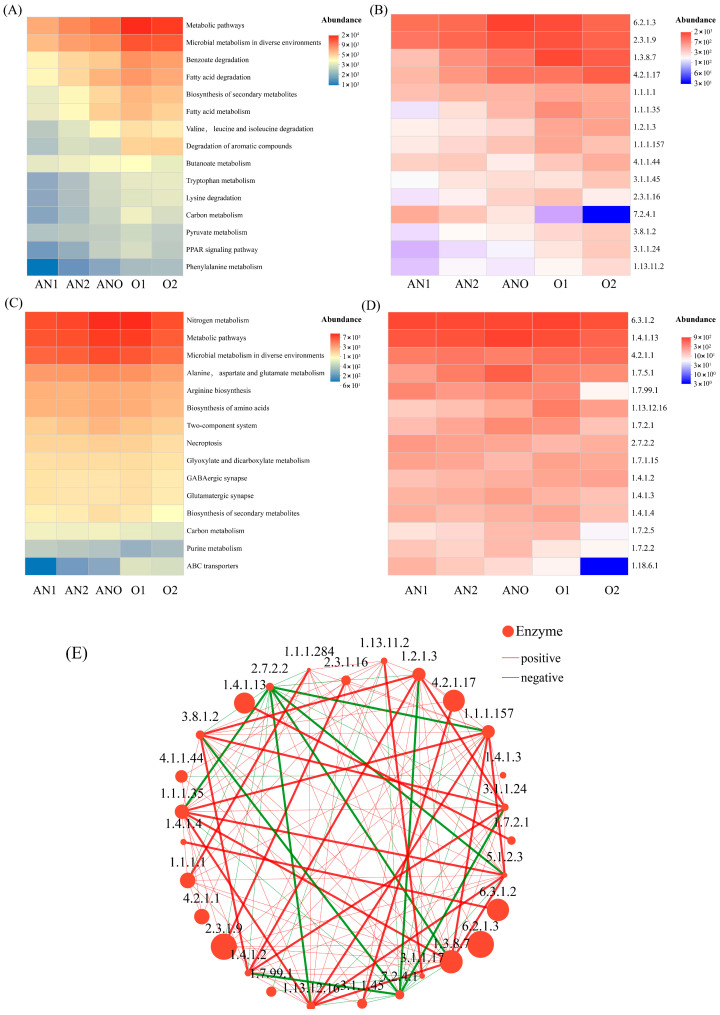
Degradation pathways and key enzymes for PHs and nitrogenous pollutants: (**A**) top 15 KEGG pathways (Level 3) involved in PH degradation; (**B**) top 15 enzymes involved in PH degradation; (**C**) top 15 KEGG pathways (Level 3) involved in nitrogen cycling; (**D**) top 15 enzymes involved in nitrogen cycling; (**E**) co-occurrence network of the top 30 enzymes based on abundance.

The primary pollutants in the produced water included residual petroleum hydrocarbons (PHs) and nitrogen-containing polymers. Key metabolic pathways involved in the degradation of PHs and nitrogenous pollutants (ko00071, ko00361, ko00362, ko00622, ko00623, ko00624, ko00625, ko00626, ko01220, and ko00910) were identified based on a review of the literature [65,66]. These pathways were subsequently used to elucidate the functional succession of microbial communities across various compartments of the bioreactor. As illustrated in Figure 6A,B, the predominant Level 3 KEGG pathways associated with PH degradation included “Metabolic pathways,” “Microbial metabolism in diverse environments,” “Benzoate degradation,” “Fatty acid degradation,” and “Biosynthesis of secondary metabolites.” The relative abundance of these functional pathways increased progressively along the treatment gradient (anaerobic → anoxic → aerobic). Analysis of key functional enzymes revealed that long-chain-fatty-acid CoA ligase (EC 6.2.1.3) and acetyl-CoA C-acetyltransferase (EC 2.3.1.9) were ubiquitously distributed, mediating the degradation of long-chain fatty acids and benzoate derivatives across all compartments. Carboxybiotin decarboxylase (EC 7.2.4.1) and carboxymuconolactone decarboxylase (EC 4.1.1.44) were predominantly enriched in the anaerobic unit. The carboxymuconolactone decarboxylase (EC 4.1.1.44) facilitated the cleavage of C–C, C–O, and C–N bonds via lyase activity to support the anaerobic degradation of aromatic and benzoate compounds. Furthermore, medium-chain acyl-CoA dehydrogenase (EC 1.3.8.7) and enoyl-CoA hydratase (EC 4.2.1.17) were primarily present in the aerobic compartments, catalyzing fatty acid oxidation reactions. Recent studies have highlighted that petroleum hydrocarbons can significantly impact microbial nitrogen cycling, while core nitrogen-cycling microorganisms, in turn, contribute reciprocally to hydrocarbon degradation through bidirectional metabolic interactions [66,67,68].

As shown in Figure 6C,D, nitrogen-cycling-related metabolic pathways in the composite bioreactor were dominated by “Nitrogen metabolism,” “Metabolic pathways,” and “Microbial metabolism in diverse environments.” Long-chain-fatty-acid CoA ligase (EC 6.2.1.3) remained highly abundant within the ko00910 nitrogen cycle pathway, indicating a strong metabolic linkage between PH degradation and nitrogen transformation processes. Carbonic anhydrase (EC 4.2.1.1), functioning as a lyase, contributed to biological nitrogen metabolism via carbon–oxygen bond cleavage and hydrolytic activity. Nitrogenase (EC 1.18.6.1), primarily detected in the anaerobic compartments, played a dual role in facilitating both nitrogen metabolism and the degradation of chlorinated alkanes/alkenes.

A co-occurrence network analysis of predicted enzyme-coding genes was conducted to further elucidate the potential roles of specific enzymes in PH biodegradation and nitrogen cycling (Figure 6E). The network comprised 28 nodes, with clustering coefficient analysis identifying key enzymes, including long-chain-fatty-acid CoA ligase (EC 6.2.1.3), enoyl-CoA hydratase (EC 4.2.1.17), carboxymethylenebutenolidase (EC 3.1.1.45), and hydroxylamine reductase (EC 1.7.99.1). These enzymes primarily mediated the hydrolysis and cleavage of molecular bonds in organic pollutants and served as the primary functional enzymes in organic degradation.

To further elucidate the keystone enzymes mediating microbial coordination in organic pollutant degradation, the centrality metrics (degree centrality, closeness centrality, and betweenness centrality) were evaluated. The result revealed that aldehyde dehydrogenase (EC 1.2.1.3), carboxybiotin decarboxylase (EC 7.2.4.1), (S)-2-haloacid dehalogenase (EC 3.8.1.2), 3-oxoadipate enol-lactonase (EC 3.1.1.24), and glutamate dehydrogenase (EC 1.4.1.2) acted as pivotal hubs that bridge multiple nodes. These keystone enzymes functioned as critical connectors in the interactive networks, thereby playing critical roles in the synergistic degradation of petroleum hydrocarbons and nitrogenous contaminants.

### 3.4. Redox Transformation Analysis of Fe^3+^/Fe^2+^

**Figure 7:**
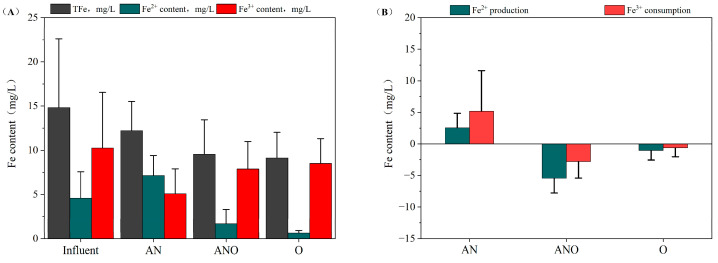
Variations in total iron, Fe^3+^, and Fe^2+^ concentrations: (**A**) concentration profiles of total iron, Fe^3+^, and Fe^2+^ across bioreactor compartments; (**B**) Fe^3+^ consumption and Fe^2+^ production.

To elucidate the redox transformation dynamics of Fe^3+^/Fe^2+^ within anaerobic compartments, Fe^2+^ production and Fe^3+^ consumption were quantified as key performance indicators (Figure 7B). As shown in Figure 7B, Fe^2+^ generation and Fe^3+^ consumption predominantly occurred in the anaerobic zones, with average values of 2.55 ± 2.30 mg/L and 5.17 ± 6.43 mg/L, respectively. This finding demonstrates that FeRB utilized electrons derived from organic matter oxidation to reduce Fe^3+^ to Fe^2+^ under anaerobic conditions, leading to progressive Fe^2+^ accumulation. Notably, the production rate of Fe^2+^ and the consumption of Fe^3+^ revealed a stoichiometric discrepancy over the operational period. Specifically, Fe^2+^ accumulation in the anaerobic compartment (AN) reached 2.55 mg/L, while net Fe^2+^ consumption occurred in the anoxic (−5.46 mg/L) and aerobic (−1.05 mg/L) units. Correspondingly, Fe^3+^ depletion was most evident in the anaerobic phase (5.17 mg/L), with substantially lower values measured in the anoxic (−2.80 mg/L) and aerobic (−0.63 mg/L) zones. These results clearly indicate that Fe^2+^ generation and Fe^3+^ reduction were predominantly confined to the anaerobic reactors. The observed stoichiometric discrepancy can be primarily attributed to the biofilm-mediated retention of Fe^2+^, as a significant fraction of the produced Fe^2+^ was adsorbed onto biofilm surfaces, thereby reducing its detectable concentration in the effluent. Additionally, microenvironmental heterogeneity within the biofilm facilitated the formation of local anaerobic microniches, enabling partial Fe^3+^ reduction by FeRB and leading to trace levels of Fe^2+^ in the aerobic effluent. Importantly, FeRB continuously oxidized organic substrates to capture electrons, thereby sustaining multi-stage organic removal through sequential iron redox cycling and microbial metabolic cascades.

### 3.5. Mechanistic Insights into Fe^3+^-Augmented Synergistic Degradation of Organic Pollutants

**Figure 8:**
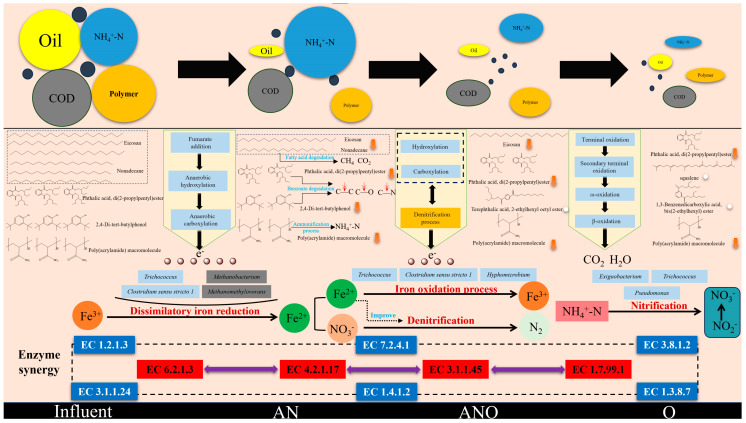
Proposed mechanism underlying Fe^3+^-enhanced degradation of organic pollutants.

Based on the integrated analysis of Fe^3+^/Fe^2+^ redox transformation and contaminant degradation (Figure 2), a comprehensive model of Fe^3+^-mediated synergistic degradation pathways was proposed (Figure 8). Under Fe^3+^ supplementation, synergistic interactions between FeRB (including *Trichococcus*, *Bacillus*, and *Clostridium sensu stricto 1*) and methanogenic archaea (including *Methanothrix*, *Methanobacterium*, and *Methanomethylovorans*) markedly facilitated the oxidation of organic contaminants. The underlying mechanisms involved dissimilatory iron reduction, maleate addition, anaerobic hydroxylation, and carboxylation processes [69,70,71]. Within the anaerobic compartments, the breakdown of long-chain alkanes, aromatic hydrocarbons, and polyacrylamides was facilitated by the cleavage of C–C, C–O, and C–N bonds through metabolic pathways such as long-chain fatty acid degradation, benzoate catabolism, and ammonification. Fe^3+^ reduction (Fe^3+^ + e^−^ → Fe^2+^) and amine dealkylation (R–NH_2_ → NH_4_^+^-N) were identified as the predominant reactions, jointly mediating the degradation of recalcitrant organic compounds. Microbial oxidation of organic substrates provided a continuous electron flow that enabled the simultaneous degradation of contaminants and biological nitrogen removal. In chemical-flooding-produced water, organic pollutants such as residual petroleum hydrocarbons and polymers act as primary electron donors. These substrates underwent progressive oxidation by hydrolytic-acidifying microorganisms, iron-reducing bacteria (FeRB), and denitrifying bacteria, during which electrons were released. These electrons served as reducing equivalents that enabled the parallel reduction of Fe^3+^ to Fe^2+^ and the stepwise reduction of nitrate and nitrite during denitrification. In the aerobic compartments, residual organic compounds underwent further transformation through mono-terminal oxidation, di-terminal oxidation, sub-terminal oxidation, ω-oxidation, and β-oxidation, while concurrent nitrification ensured a sustained supply of NO_3_^−^, reinforcing denitrification and contaminant removal in the anoxic environment. Under Fe^3+^-enriched conditions, the collective action of multiple bioactive enzymes played a decisive role in the bioconversion of organic pollutants in produced water. Notably, auxiliary enzymes (e.g., EC 1.2.1.3, EC 7.2.4.1, EC 3.8.1.2, EC 3.1.1.2, and EC 1.4.1.2) enhanced the catalytic activity of primary degradative enzymes (EC 6.2.1.3, EC 4.2.1.17, EC 3.1.1.45, and EC 1.7.99.1), collectively driving the bio-oxidation and mineralization of complex organics.

## 4. Conclusions

This study comprehensively investigated the performance and microbial community dynamics of an Fe^3+^-augmented composite bioreactor for the treatment of oilfield-produced water. Fe^3+^ supplementation, as a bioenhancement strategy, not only significantly improved the metabolic activity and syntrophic interactions of key functional microorganisms (e.g., FeRB and methanogenic archaea) in the anaerobic zones but also facilitated the efficient biodegradation of recalcitrant organic pollutants such as petroleum hydrocarbons and polymers. The anaerobic compartments, serving as the core biotreatment module in the composite bioreactor, achieved high removal efficiencies for oil (96.22%), polymers (48.49%), and COD (39.45%). Microbial community analysis revealed a pronounced enrichment of FeRB (e.g., *Trichococcus* and *Bacillus*) and their synergistic interactions with methanogenic archaea (e.g., *Methanobacterium* and *Methanomethylovorans*), underscoring the importance of microbial syntrophy in multi-pollutant removal under Fe^3+^ enhancement. Furthermore, functional gene prediction identified key enzymes—including long-chain-fatty-acid CoA ligase (EC 6.2.1.3), enoyl-CoA hydratase (EC 4.2.1.17), carboxymethylenebutenolidase (EC 3.1.1.45), and hydroxylamine reductase (EC 1.7.99.1)—that played central roles in the biodegradation of petroleum hydrocarbons and nitrogenous polymers. This study provides novel insights into the dynamic regulation of the microbial community structure and function under Fe^3+^ bioaugmentation in a composite bioreactor and elucidates a multi-mechanistic pathway for the synergistic degradation of refractory organic pollutants. The findings provide a technical basis for the development of green, efficient, and sustainable produced-water treatment solutions in the oil industry.

## Figures and Tables

**Table 1:** Physicochemical properties of the oilfield-produced water.

Parameter	Value
COD (mg/L)	492.69 ± 192.03
Oil content (mg/L)	129.63 ± 92.57
SS content (mg/L)	144.62 ± 44.29
Polymer concentration (mg/L)	42.51 ± 13.24
NH_4_^+^-N (mg/L)	15.17 ± 3.30
Viscosity (mPa∙s)	0.70 ± 0.03
pH	7.56 ± 0.05

**Table 2:** Bacterial diversity indices of sludge samples.

Sample	Ace	Chao	Coverage	Shannon	Simpson	Sobs
AN1	1416	1412	0.9939	3.51	0.17	1156
AN2	1516	1506	0.9941	4.16	0.12	1259
ANO	1607	1615	0.9930	3.59	0.21	1283
O1	1555	1557	0.9933	4.38	0.05	1254
O2	1091	1076	0.9958	4.24	0.04	913

## Data Availability

The original contributions presented in this study are included in the article; further inquiries can be directed to the corresponding authors.
